# *Leishmania* carbon metabolism in the macrophage phagolysosome- feast or famine?

**DOI:** 10.12688/f1000research.6724.1

**Published:** 2015-10-01

**Authors:** Malcolm J. McConville, Eleanor C. Saunders, Joachim Kloehn, Michael J. Dagley

**Affiliations:** 1 Department of Biochemistry and Molecular Biology, Bio21 Molecular Science and Biotechnology Institute, University of Melbourne, Flemington Rd, Parkville, 3010, Australia

**Keywords:** central carbon metabolism, virulence, reductive stress, macrophages, Leishmania

## Abstract

A number of medically important microbial pathogens target and proliferate within macrophages and other phagocytic cells in their mammalian hosts. While the majority of these pathogens replicate within the host cell cytosol or non-hydrolytic vacuolar compartments, a few, including protists belonging to the genus *Leishmania*, proliferate long-term within mature lysosome compartments.  How these parasites achieve this feat remains poorly defined. In this review, we highlight recent studies that suggest that *Leishmania* virulence is intimately linked to programmed changes in the growth rate and carbon metabolism of the obligate intra-macrophage stages. We propose that activation of a slow growth and a stringent metabolic response confers resistance to multiple stresses (oxidative, temperature, pH), as well as both nutrient limitation and nutrient excess within this niche. These studies highlight the importance of metabolic processes as key virulence determinants in *Leishmania*.

## Introduction

Macrophages play key roles in the mammalian innate and adaptive immune responses ^1^. These cells are actively recruited to sites of tissue damage and infection and are able to kill a wide range of invading bacterial, fungal, and protozoan pathogens following phagocytosis and their delivery to the lysosome compartment ^1^. Not surprisingly, a number of medically important microbial pathogens have developed strategies to either avoid phagocytosis by macrophages or to subvert uptake into the mature lysosome compartment. The latter group either prevent maturation of the phagosomes within which they are internalized or escape into the cytosol, or both (for example, *Mycobacterium tuberculosis*, *Salmonella spp.*, *Trypanosoma cruzi*) ^2, 3^. Other pathogens invade macrophages via phagocytosis-independent mechanisms and reside within non-hydrolytic compartments in these cells (for example, *Toxoplasma gondii*) ^4^. However, a small number of pathogens are internalized into the mature phagolysosome compartment of macrophages and are capable of long-term survival and proliferation within this compartment ^5– 7^. These include the protozoan parasites belonging to the genus *Leishmania* which in humans cause a spectrum of diseases ranging from localized cutaneous skin lesions to disseminating mucocutaneous infections and deadly visceral infections ^7^. Strikingly, mammalian-infective stages of *Leishmania* lack many of the conventional virulence determinants of other pathogens, such as a thick cell wall, or cytoprotective pigments, suggesting that they may be more dependent on physiological changes. Although some progress has been made in identifying signaling pathways and other processes that are important for *Leishmania* virulence in the mammalian host ^6, 8– 11^, major gaps in our understanding of *Leishmania* amastigote survival strategies remain. Here, we summarize recent studies that suggest that intracellular survival is linked to a marked decrease in parasite growth and a rewiring of central carbon metabolism. These changes may underlie the intrinsic resistance of these parasite stages to many stresses (temperature, pH) and their tolerance of both nutrient limitation and nutrient excess (feast and famine) in this intracellular niche.

## Living in the macrophage phagolysosome

**Figure 1.**
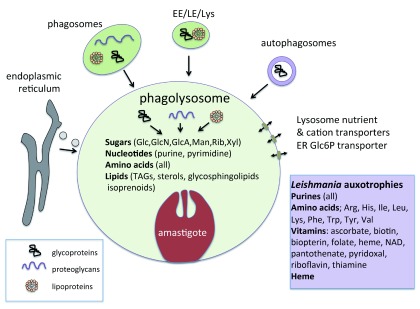
*Leishmania* replicate within the mature phagolysosome compartment of macrophages. This compartment is predicted to contain a range of carbon sources (sugars, amino acids, and fatty acids) and essential nutrients (major auxotrophic requirements listed in insert) that are delivered to the phagolysosome via different endocytic pathways, autophagy, lysosomal membrane transporters, and fusion with the endoplasmic reticulum (ER). Macromolecules delivered to this compartment are degraded by a barrage of luminal hydrolases or internalized by amastigotes and degraded within their own hydrolytically active lysosomes, or both. Arg, arginine; EE, early endosome; Glc, glucose; Glc6P, glucose 6-phosphate; GlcA, glucuronic acid; GlcN, glucosamine; His, histidine; Ile, isoleucine; LE, late endosome; Leu, leucine; Lys, lysine; Man, mannose; Phe, phenylalanine; Rib, ribose; TAG, triacylglycerol; Trp, tryptophan; Tyr, tyrosine; Val, valine; Xyl, xylose.

*Leishmania* spp. develop as flagellated promastigotes in the lumen of their sandfly vectors and are transmitted to a range of human and animal hosts when the sandfly takes a bloodmeal. After injection into the skin, promastigotes are initially internalized by neutrophils before being phagocytosed by macrophages and delivered to the mature phagolysosome compartment where they differentiate to the small, round, aflagellate amastigote stage ^6^. The further recruitment of macrophages to the site of infection results in the formation of lesions or granuloma-like structures that are the hallmark of all *Leishmania* infections ^11, 12^. Macrophages are the predominant cell type within lesions and can be infected with a few to several hundred amastigotes that, depending on the species involved, reside either within individual tight-fitting vacuoles (one parasite per vacuole) or within large spacious communal vacuoles. These vacuoles have a low pH (~5.4) and contain all of the membrane and luminal markers of a mature phagolysosome, including the characteristic suite of hydrolases and the membrane NADH oxidase that generates anti-microbial oxidative burst. The *Leishmania*-occupied phagolysosome compartment appears to be highly dynamic, receiving a wide range of host macromolecules via fusion with vesicles from the phagocytic, endocytic, and autophagic pathways as well as the endoplasmic reticulum ( Figure 1) ^6, 13^. These macromolecules are degraded by luminal hydrolases (proteases, lipases, glycosidases) to generate free sugars, lipids, and peptides/amino acids which can be taken up by amastigote plasma membrane transporters. Amastigotes can also internalize host macromolecules directly and degrade many of them within their own lysosome. Thus, the phagolysosome compartment may contain a wide array of carbon sources and essential nutrients, in contrast to other compartments in the endo-lysosomal network ^14^. Consistent with this notion, *Leishmania* are auxotrophic for many essential nutrients, including purines, vitamins, heme, and a range of amino acids, which must be scavenged from the lysosome. Similarly, a number of *Leishmania* mutants have been generated with defects in pathways for *de novo* synthesis of other metabolites (glycine, amino sugars) or nutrient salvage pathways (nucleotide/nucleoside/purine base) that retain virulence in animal models, suggesting considerable redundancy in nutrient uptake/ *de novo* biosynthetic pathways ^15– 18^. Indeed, we have previously proposed that the complex auxotrophic requirements of these parasites may underlie their tropism for this intracellular niche ^6^ ( Figure 1). Interestingly, the Gram-negative bacterium *Coxiella burnetii*, one of the few other microbial pathogens to survive long term within in the macrophage phagolysosome, exhibits a similar broad range of nutrient auxotrophies ^19^. Therefore, the macrophage phagolysosome may represent a relatively permissive intracellular niche with regard to nutrient availability, if microbes can establish suitable strategies for inhibiting or evading the activation of highly effective host cell microbiocidal processes.

## *Leishmania* amastigotes enter a quiescent state and exhibit a stringent metabolic response

Up until recently, information on the growth rate and metabolic state of *Leishmania* amastigotes in inflammatory lesions and granulomas was limited. A number of studies have tracked changes in *Leishmania* parasite load in both susceptible and resistant murine models by monitoring changes in parasite numbers or by following transgenic parasites lines expressing luciferase or fluorescent reporter proteins ^20– 23^. These studies suggest that *Leishmania* amastigotes undergo progressive and continuous replication in susceptible mice strains (such as BALB/c), leading to systemic infection and death. In contrast, while parasite numbers increase in resistant mice strains (such as C57BL/6) during early stages of infection, numbers subsequently plateau and eventually are reduced to a low level as a protective host immune response develops. Thus, net changes in *Leishmania* parasite burden are determined by both parasite growth rate and the rate of parasite clearance or dissemination to other tissues (or both), which will vary with the immune status of the host. Recently, two distinct approaches have been developed to more precisely determine both the growth rate and metabolic state of *Leishmania* amastigotes *in vivo*. In the first approach, transgenic *L. major* lines were generated expressing a photo-convertible fluorescent protein and used to monitor both amastigote dissemination in inflammatory lesions and overall protein turnover as a proxy of their growth and metabolic state ^24^. This study showed that there was very little migration of *L. major* amastigote-infected macrophages into or out of these lesions and that intracellular amastigotes exhibited surprisingly low rates of protein turnover and, by inference, replication. Interestingly, the slow rate of parasite replication in these tissues appeared to reflect, at least partially, the production of sub-lethal concentrations of nitric oxide by lesion macrophages ^24^.

In the second approach, the growth rate of *L. mexicana* amastigotes in inflammatory lesions in susceptible BALB/c mice was measured by labeling infected mice with heavy water ( ^2^H _2_O) ^25^. ^2^H _2_O labeling results in the incorporation of deuterium into a wide range of metabolic precursors in both host tissues and resident parasite populations, and the subsequent incorporation of these building blocks into macromolecules can be used to determine the turnover of key cellular components (DNA, RNA, proteins, and lipids). With this novel approach, *L. mexicana* amastigotes were found to divide at a very slow, but constant, rate (t _1/2_ ~12 days on the basis of DNA turnover) throughout lesion development ^25^. The growth rate of lesion parasites was substantially slower than in cultured macrophages, supporting the notion that parasite growth in lesions is constrained, by either autonomous or host-microbicidal responses. Furthermore, the empirically determined amastigote growth rates closely matched those calculated from overall parasite burden (total parasites and parasites per macrophage), suggesting that parasite killing in BALB/c lesions is rare and that infected lesion macrophages are very long-lived. The ^2^H _2_O labeling approach was further extended to measure global rates of RNA and protein turnover in lesion amastigotes ^25^. Both processes were found to be repressed to a greater extent than in non-dividing insect (promastigote) stages, suggesting that lesion amastigotes enter into a semi-quiescent state in which major energy-consuming processes are specifically repressed.

**Figure 2.**
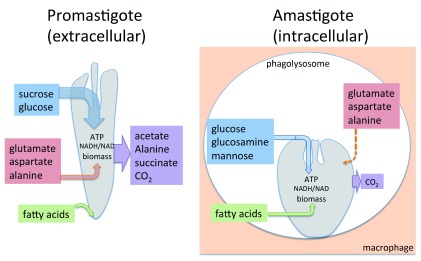
Intracellular amastigotes exhibit a stringent metabolic response. The differentiation of *Leishmania* promastigotes (insect stage) to amastigotes (macrophage host) is associated with major changes in central carbon metabolism. Promastigotes exhibit high rates of glucose and (non-essential) amino acid uptake that are co-catabolized via the major pathways of central metabolism. Promastigotes also take up fatty acids, but these are primarily incorporated into membrane lipids and not used as carbon sources (downward arrow). Amastigotes also preferentially use glucose as a carbon source. However, they exhibit much lower (~10-fold) rates of sugar and amino acid uptake and overflow metabolism (note that amastigotes continue to take up essential amino acids but primarily use these for protein synthesis). Amastigotes also actively catabolize fatty acids in the tricarboxylic acid (TCA) cycle, as a result of reduced glucose uptake. The downregulation of hexose/amino acid uptake in amastigotes (stringent response) is hardwired to differentiation, as it occurs *in vitro* irrespective of nutrient levels and is coupled to a reduced growth rate ^27^.

Metabolite profiling and ^13^C-stable isotope labeling of isolated lesion amastigotes have suggested that entry into this metabolically quiescent state is associated with major rewiring of key fluxes in central carbon metabolism ^26, 27^. In particular, lesion amastigotes have dramatically reduced rates of glucose and amino acid uptake and use these carbon sources much more efficiently than rapidly replicating or non-dividing promastigotes ^27^ ( Figure 2). Specifically, both dividing and non-dividing promastigote stages take up more glucose than is needed to maintain or increase biomass and exhibit high levels of overflow metabolism (secretion of partially oxidized glucose end-products, such as acetate, succinate, and alanine). In contrast, amastigotes exhibit much reduced rates of glucose uptake but negligible rates of overflow metabolism (glucose-sparing) ( Figure 2). This switch to a more economical metabolism in amastigotes has been termed the stringent response and is associated with reduced uptake of other potential carbon sources, such as amino acids ^27^. This response appears to be hard-wired into the amastigote differentiation process as a similar downregulation of glucose and amino acid uptake also occurs in *in vitro* differentiated amastigotes regardless of the availability of glucose or other carbon sources in the medium.

How this stage-specific switch in metabolism is regulated remains largely undefined. *Leishmania* are unusual in lacking conventional gene-specific transcriptional regulation (and transcription factors) and constitutively transcribe gene-rich regions of their genome as long polycistronic mRNAs that are subsequently processed to generate individual mRNA ^28^. As a result, the levels of most protein-encoding mRNAs remain constant in both dividing and non-dividing developmental stages ^29^. Similarly, most metabolic enzymes are constitutively expressed and any stage-specific differences in protein levels, where present, are modest (generally less than a twofold) or variable (or both) across different *Leishmania* species ^30, 31^. Post-translational mechanisms are therefore likely to play an important role in the induction of the amastigote stringent response. There is accumulating evidence that several key nutrient transporters involved in glucose and amino acid uptake are downregulated in amastigotes. In the case of glucose transporters, downregulation can be mediated by ubiquitination of the cytoplasmic tail and internalization and degradation of the transporter in the parasite lysosome ^32^. Ubiquitination or sumoylation has also been shown to regulate key pathways, such as fatty acid β-oxidation ^33^. The upstream signals and processes that trigger these changes are poorly defined. However, amastigote differentiation is associated with marked changes in the phosphorylation state of many proteins, including those involved in stress responses ^34^, and several protein kinases ^8, 9^ and phosphatases ^8– 10, 35^ have been shown to be essential for virulence, suggesting that different signaling cascades may be required for the activation of the stringent response.

## What is the function of metabolic quiescence?

The finding that *Leishmania* amastigotes enter a slow growth/metabolically quiescent state was unexpected given the available evidence suggesting that the phagolysosome compartment contains a variety of potential carbon sources. One explanation for this apparent paradox is that the phagolysosome, while containing high levels of some carbon sources, may be growth-limiting with regard to the availability of other (micro)nutrients. Consistent with this notion, intracellular amastigote growth in *ex vivo* infected macrophages and *in vivo* is promoted by increasing the availability of select amino acids, such as arginine ^36– 41^. In the latter case, it remains unclear whether promotion of amastigote growth is due to increased availability of arginine, an essential amino acid, or conversion of arginine to growth-promoting polyamines by the host cell arginase. Moreover, active salvage of arginine by intracellular parasites may deplete arginine pools in the macrophage and affect the capacity of these host cells to generate nitric oxide via inducible nitric oxide synthase ^41^, further complicating the interpretation of these supplementation experiments. Similarly, there is strong evidence that phagolysosomal levels of micronutrients, such as iron and heme, can regulate intracellular parasite growth ^42– 46^. Host cell transporters in the macrophage phagolysosomal membrane pump iron and heme out of the phagolysosome lumen to the cytosol and thus are important determinants of amastigote growth ^42, 43, 47– 49^. In response, *Leishmania* amastigotes upregulate expression of a surface ferric reductase (that converts Fe ^3+^ to Fe ^2+^) and a ferrous (Fe ^2+^) iron transporter, LIT1, allowing efficient salvage of iron, an essential cofactor in many parasite enzymes, including the parasite iron superoxide dismutase (FeSOD) and iron-sulphur containing enzymes involved in the mitochondrial tricarboxylic acid (TCA) cycle. Interestingly, *L. amazonensis* mutants that lack the LIT1 transporter are unable to retain viability when promastigote stages reach stationary phase or to effectively differentiate to amastigotes ^43^. Differentiation was found to be dependent on FeSOD-mediated conversion of superoxide to hydrogen peroxide, which appears to stimulate amastigote differentiation. Thus, iron restriction within the phagolysosome may have a dual effect of preventing induction of the stringent response as well as limiting operation of energy-generating pathways in the mitochondria. Together, these studies suggest that selected nutrient restriction occurs in the phagolysosome and that *Leishmania* adapt to this niche by upregulating the expression of specific nutrient sensing and salvage pathways as well as downregulating global energy requirements (stringent response).

The *Leishmania* amastigote stringent response is induced in response to elevated temperature and reduced pH in culture, suggesting that these physiological changes may protect parasites from these specific environmental stresses or that it is part of a programmed stress response to multiple stresses (that can be triggered by these key signals *in vivo*) or both. In support of the latter proposal, the stringent response is enhanced in lesion amastigotes compared with cultured (axenic) amastigotes. As mentioned above, amastigote growth in developing lesions may be restricted by sublethal concentrations of reactive nitrogen species (RNS), which can inactivate many enzymes in the mitochondrial TCA cycle and respiration chain containing iron-sulphur clusters ^50^. A switch to increased dependency on glycolysis and an overall reduction in basal energetic requirements would reduce amastigote vulnerability to macrophage-derived RNS. Interestingly, a number of other bacterial pathogens that invade macrophages also appear to be dependent on sugars as their major carbon source ^51^, and decreased bacterial respiration is associated with resistance to a range of external stresses, including microbicidal NO and drug treatments ^52^.

**Figure 3.**
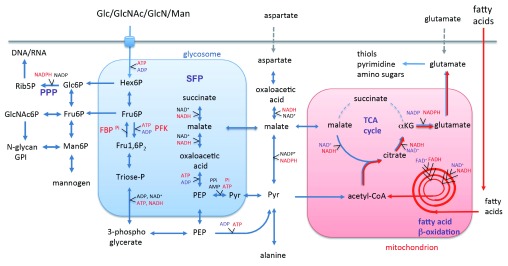
Carbon metabolism of *Leishmania* amastigotes. *Leishmania* amastigotes appear to depend primarily on the uptake and catabolism of sugars scavenged from the macrophage phagolysosome. Hexose phosphates are catabolized in the glycolytic and pentose phosphate pathway (PPP) and converted to intracellular and surface glycoconjugates (GPI, N-glycans, mannogen). Key enzymes involved in glycolysis are partially or exclusively sequestered within glycosomes (modified peroxisomes), and ATP and NAD ^+^ within this organelle are regenerated by fermentation of phosphoenolpyruvate to succinate (succinate fermentation pathway, or SFP) or pyruvate ^66^. The end-products of glycosomal catabolism are further catabolized in the mitochondrion, together with acetyl-CoA generated by fatty acid β-oxidation, to produce anabolic precursors, such as glutamate. Most of the glutamate (and other non-essential amino acids) in amastigotes is synthesized *de novo* rather than taken up from macrophages. Excess NADH production in the mitochondrion might lead to increased endogenous reactive oxygen species (ROS) production via the respiratory chain. The gluconeogenic enzyme, fructose-1,6-bisphosphatase (FBP), is also required for amastigote survival *in vivo*. This enzyme is sequestered in glycosomes with phosphofructokinase (PFK) and might allow amastigotes to transiently use other carbon sources or regulate glycolytic fluxes by cycling FBP back to fructose 6-phosphate (futile cycling), or both. αKG, α-ketoglutarate; AcCoA, acetyl-CoA; Fru6P, fructose-6-phosphate; Glc6P, glucose-6-phosphate; GlcNAc6P, N-acetylglucosamine-6-phosphate; Glu, glutamate; Man6P, mannose-6-phosphate; PEP, phosphoenolpyruvate; Pyr, pyruvate; Rib5P, ribose-5-phosphate; Triose-P, triose phosphates.

The stringent response may also protect amastigotes from nutrient excess. The concept that nutrient excess can lead to cellular stress is now well established in diseases such as obesity, metabolic syndrome, and diabetes ^53, 54^ but less commonly considered in microbes, particularly those in intracellular niches ^55^. Metabolic stress induced by nutrient overload (that is, excess glucose) can occur as a result of multiple mechanisms, of which the most prevalent are increased production of mitochondrial NADH (that is, increased NADH/NAD ^+^ ratio) and concomitant elevated production of endogenous reactive oxygen species (ROS) as a result of leakage of electrons from the mitochondrial respiratory chain ^53^. *Leishmania* are potentially highly vulnerable to reductive stress, as they lack the capacity to transcriptionally downregulate TCA cycle enzymes involved in NADH generation and, owing to the compartmentalization of glycolytic enzymes into modified peroxisomes, termed glycosomes, also appear to have lost classic allosteric regulatory mechanisms that result in feedback inhibition of glycolysis ^6^ ( Figure 3). The absence of allosteric feedback mechanisms in upper glycolysis means that glycolytic fluxes are largely regulated by glucose uptake rates. *Leishmania* promastigotes can exploit high concentrations of glucose and avoid excessive flux into the TCA cycle (with concomitant NADH production) by secreting partially oxidized intermediates, such as alanine, acetate, and succinate into the medium ^26^ ( Figure 2). A similar strategy is used by other microorganisms, such as *Saccharomyces cerevisiae*, during periods of rapid growth on fermentable carbon sources ^56^. However, the profligate use of carbon sources and secretion of partially oxidized intermediates is likely to be deleterious for intracellular parasite stages and could also impact on host cell physiology. The global downregulation of amastigote nutrient transporters after activation of the stringent response ^32^ may constitute an important strategy for minimizing nutrient uptake and reductive stress within the restrictive environment of the phagolysosome ^32^.

## Rewiring of carbon metabolism may also be used to deal with nutrient excess?

Activation of the *Leishmania* stringent response in amastigotes is linked to additional changes in carbon metabolism that could also contribute to parasite survival within macrophages. Detailed ^13^C-tracer studies ^27^ have shown that lesion amastigotes, in common with promastigote, appear to preferentially use sugars, although rates of uptake are much lower than in promastigotes. Whereas most compartments within the endolysosomal system of macrophages are thought to contain low luminal concentrations of sugars, the (phago)lysosome compartment may be an exception. Macrophages constitutively internalize a wide range of complex glycoproteins, proteoglycans, and glycosaminoglycans that are degraded by lysosomal glycosidases to generate free sugars or oligosaccharides. *Leishmani*a hexose transporters ^57^ and enzymes involved in the catabolism of host-derived amino sugars are essential for *Leishmania* virulence ^58, 59^. Furthermore, intracellular growth of *Leishmania* amastigotes in cultured macrophages can be stimulated by the addition of glycosaminoglycans such as hyaluronan, highlighting the importance of amino sugar catabolism for *Leishmania* survival and virulence ^58, 59^.

Amastigotes also co-utilize fatty acids as a significant carbon source ( Figure 3). This contrasts with promastigotes that preferentially co-utilize non-essential amino acids, aspartate, alanine, and glutamate with glucose ^26, 27^. The increased β-oxidation of fatty acids in amastigotes appears to be a direct consequence of reduced glucose uptake by this stage ^27^, and the resultant acetyl-CoA produced by fatty acid oxidation is used primarily to top up the TCA cycle (anapleurosis) providing intermediates for the biosynthesis of amino acids, such as glutamate, glutamine, and aspartate (catapleurosis) ( Figure 3). Pharmacological inhibition of enzymes involved in the synthesis of non-essential amino acids via the TCA cycle results in complete inhibition of amastigote growth and survival ^27^. These amino acids are required for nucleotide, thiol and amino-sugar biosynthesis, and the dependence on *de novo* synthesis is consistent with the finding that amino acid uptake by amastigotes is limited ^57, 59, 60^. Similarly, genetic disruption of fatty acid β-oxidation or proteins involved in the mitochondrial respiratory chain also results in a loss of virulence ^33, 61^. Together, these studies suggest that amastigotes are highly dependent on sugar and fatty acids scavenged from the lumen of the phagolysosome.

Paradoxically, *Leishmania* amastigote mutants lacking the key gluconeogenic enzyme, fructose 1,6-bisphosphatase (FBPase), are also poorly virulent in mice ^62^. FBPase catalyzes the conversion of fructose-1,6-bisphosphate to fructose-6-phosphate and is expressed in the same glycosome compartment as the glycolytic enzyme, phosphofructokinase (PFK), that catalyzes the reverse reaction ^62^ ( Figure 3). The functional significance of the constitutive expression of these two enzymes in the same organelle remains unclear. Sugar levels in the phagolysosome could fluctuate in response to changes in membrane transport and the delivery of cargo to this compartment, leading to periods of sugar starvation and transient dependency on gluconeogenesis for the synthesis of essential glycoconjugates, DNA/RNA synthesis, and production of reducing equivalents via the pentose phosphate pathway ^63^. In this context, co-expression of both FBPase and PFK could allow *Leishmania* amastigotes to rapidly respond to changes in carbon source availability. However, lesion-derived amastigotes exhibit very low rates of amino acid uptake and intracellular stages appear to be dependent on glucose catabolism even when infected macrophages are supplied with excess amino acids ^27^. Furthermore, *Leishmania* lack a glyoxylate cycle and therefore are unable to switch to using fatty acids (a likely plentiful carbon source in this compartment) as a sole gluconeogenic carbon source. It is possible that FBPase may have acquired non-enzymatic functions, other than its role in gluconeogenesis, that account for the dependency of intracellular stages on this enzyme. FBPase has recently been shown to regulate glycolysis in mammalian cells via at least two mechanisms, one of which involves transcriptional regulation of signaling proteins and is not dependent on its enzymatic activity ^64^. Alternatively, FBPase may be required for parasite growth under growth conditions in which glucose uptake and glycolysis are still active ( Figure 3). This has recently been shown to be the case in *Toxoplasma gondii*, another intracellular parasite that resides in a distinct vacuolar compartment and is also primarily dependent on glucose catabolism for growth ^65^. As with *L. major*, genetic disruption of *T. gondii* FBPase resulted in strong attenuation of intracellular growth in host cells and loss of virulence in animal models. Loss of virulence of the *T. gondii ∆FBPase* mutant was associated with increased glycolytic flux at the expense of glucose flux into other essential metabolic pathways. Thus, under normal growth conditions, *T. gondii* FBPase may function in a futile (ATP-consuming) metabolic cycle with the PFK and potentially restrict excessive flux through glycolysis and ensure balanced growth. Whether metabolic cycling between FBPase and PFK occurs in the *Leishmania* amastigote’s glycosome and the extent to which it regulates glycolytic fluxes remains to be determined.

## Conclusions

*Leishmania* parasites are unusual in their capacity to proliferate long-term within the mature phagolysosome compartment of host macrophages. It is likely that the complex nutritional requirements of *Leishmania* and the need to have access to a broad range of metabolites underlie *Leishmania*’s tropism for this hostile intracellular niche. However, successful colonization of this niche must have been linked to the parallel evolution of strategies for combating a range of host cell microbicidal processes (ROS, RNS, hydrolases) that are normally effective at eradicating pathogens that are delivered to this compartment. Intriguingly, *Leishmania* amastigotes lack many of the virulence factors found in promastigotes or other microbial pathogens (cell walls, surface coats, protective pigments, and so on), suggesting that the extraordinary resilience of these pathogens is dependent on more fundamental physiological changes that confer cytoprotection against a variety of stresses. Very recent studies, using new fluorescent protein reporters and stable isotope ( ^2^H, ^13^C) labeling approaches for measuring amastigote physiology and metabolism *in vivo*, suggest that amastigotes enter into a semi-quiescent growth state *in vivo*. This state is distinct from that observed in non-dividing promastigotes and appears to be programmed by differentiation signals independent of external nutrient levels. It is proposed that induction of the stringent metabolic response may (i) prevent depletion of essential limiting (micro)nutrients in the phagolysosome compartment, (ii) reduce the bioenergetic needs of amastigotes and hence their dependence on high-energy-yielding pathways (such as oxidative phosphorylation) that are highly susceptible to inhibition by RNS/ROS, and (iii) minimize endogenous reductive stress induced by excessive utilization of abundant carbon sources in the phagolysosome and overflow metabolism. Thus, the stringent metabolic response may protect amastigotes from both feast and famine within this compartment. Further studies are needed to understand how amastigote metabolism is regulated in the absence of significant gene-specific transcriptional regulation, while the identification of key steps in carbon metabolism that are essential for amastigote virulence opens up new opportunities for the development of novel anti-microbial strategies.

## Abbreviations

^2^H _2_O, heavy water; FBPase, fructose-1,6-bisphosphatase; FeSOD, iron superoxide dismutase; PFK, phosphofructosekinase; RNS, reactive nitrogen species; ROS, reactive oxygen species; TCA, tricarboxylic acid.
