# Supplementary material for: Leishmania carbon metabolism in the macrophage phagolysosome- feast or famine?
Source: F1000Res. 2015 Oct 1;4(F1000 Faculty Rev):938. [Version 1] doi: 10.12688/f1000research.6724.1 (PMC4648189; doi:10.12688/f1000research.6724.1)
Supplement: Peer review history

# Peer review: Referee response for version 1

**DOI:** 10.5256/f1000research.7222.r10644

**Published:** 2015-10-01

**Authors:** Norma Andrews, Maria Fernanda Laranjeira Silva

**Affiliations:** 1 Department of Cell Biology & Molecular Genetics, University of Maryland, College Park, MD, 20742, USA

We have read this submission. We believe that we have an appropriate level of expertise to confirm that it is of an acceptable scientific standard.

# Peer review: Referee response for version 1

**DOI:** 10.5256/f1000research.7222.r10643

**Published:** 2015-10-01

**Authors:** Marilyn Parsons

**Affiliations:** 1 Emerging & Neglected Disease Program, Seattle Biomedical Research Institute, Seattle, WA, 98109, USA

I have read this submission. I believe that I have an appropriate level of expertise to confirm that it is of an acceptable scientific standard.

# Peer review: Referee response for version 1

**DOI:** 10.5256/f1000research.7222.r10642

**Published:** 2015-10-01

**Authors:** Syamal Roy

**Affiliations:** 1 Infectious Diseases & Immunology Division, Indian Institute of Chemical Biology, Kolkata, West Bengal, 700 032, India

I have read this submission. I believe that I have an appropriate level of expertise to confirm that it is of an acceptable scientific standard.
